# Complete mitochondrial genome of the Mexican marine littoral hygrophilous *Spinactaletes boneti* (Collembola: Actaletidae) and its phylogenetic placement

**DOI:** 10.1080/23802359.2022.2068982

**Published:** 2022-05-03

**Authors:** Nerivania Nunes Godeiro, José G. Palacios-Vargas, Yan Gao, Yun Bu

**Affiliations:** a Shanghai Natural History Museum, Shanghai Science & Technology Museum, Shanghai, China; b Laboratorio de Ecología y Sistemática de Microartrópodos, Departamento de Ecología y Recursos Naturales, Facultad de Ciencias, Universidad Nacional Autónoma de México, México

**Keywords:** Mitogenome, Actaletidae, intertidal zone, Mexico

## Abstract

*Spinactaletes boneti* mitochondrial genome was assembled and annotated. It has 14,766 bp in length, all 37 genes are present and the gene order is the same as the Pancrustacean ancestral gene order. Phylogenetic analyses based on maximum likelihood placed the species as a sister group of the remaining Entomobryomorpha, not closely related to the Isotomoidea superfamily, contradicting the actual systematics of the group.

## Introduction

*Spinactaletes boneti* (Parisi 1972) is distributed along the intertidal zone of the Pacific Coast, from Zihuatanejo to Acapulco, Mexico. It belongs to the family Actaletidae (*Actaletes* with one species in Europe, *Spinactaletes* with 11 in the Americas). Representatives of this family can be distinguished from the other entomobryids by the presence of a tracheal system, abdomen III reduced, abdomen IV and V fused, abdomen VI reduced to the anal valves, abdomen IV with five sutures (two in the tergo, one intersegmental and two sternal), and 3–4 trichobothria. *Spinactaletes* is a genus of obligatory marine littoral springtails, mostly associated with calcareous rocks (Soto-Adames and Guillén 2011). The systematic position of the Actaletidae family is doubtful, some authors considered it close to Isotomidae/Isotomoidea (Yosii 1961; Massoud 1976; Soto-Adames 2008), to Poduridae (Paclt 1956; Salmon 1964), to Sminthuridae (Dallai & Malatesta, 1973), or as a sister group of all remaining Entomobryomorpha families (D’Haese 2003). Currently, the family is included in the Isotomoidea superfamily, because their species share a few morphological characters with some genera of Isotomoidea (Soto-Adames 2008).

The specimen of *S. boneti* sequenced here was collected from the surface of rock formations in the intertidal zone of the touristic Zihuatanejo city, Guerrero State, Mexico (17.622 N, 101.514 W) by Yun BU and José Palacios-Vargas on 10 November 2019. Twenty specimens were deposited at Shanghai Natural History Museum (Yun BU, email: buy@sstm.org.cn). One individual (voucher number MX-ZH-2019005) was used for DNA extraction and whole-genome amplification. All laboratory experiments including library construction and sequencing were performed by Shanghai Yaoen Biotechnology Co., Ltd, China. Illumina NovaSeq platform was used for sequencing paired-end reads with 150 bp length, producing approximately 10 G of data. The mitogenome was assembled de novo using NovoPlasty v3.8.3 (Dierckxsens et al. 2020) with kmer value 28 and a COI partial sequence of *Mesaphorura yosii* Yosii, 1906 was used as a seed (accession number KT799636.1). The identity and position of the 13 PCGs, 22 tRNA, and 2 rRNA genes were determined using MitoZ v2.4-alpha (Meng et al. 2019). The gene order of the new mitogenome was manually checked and it is the same as the Pancrustacean, which is the most common gene order across the Collembola group (Leo et al. 2019).

**Figure 1.**
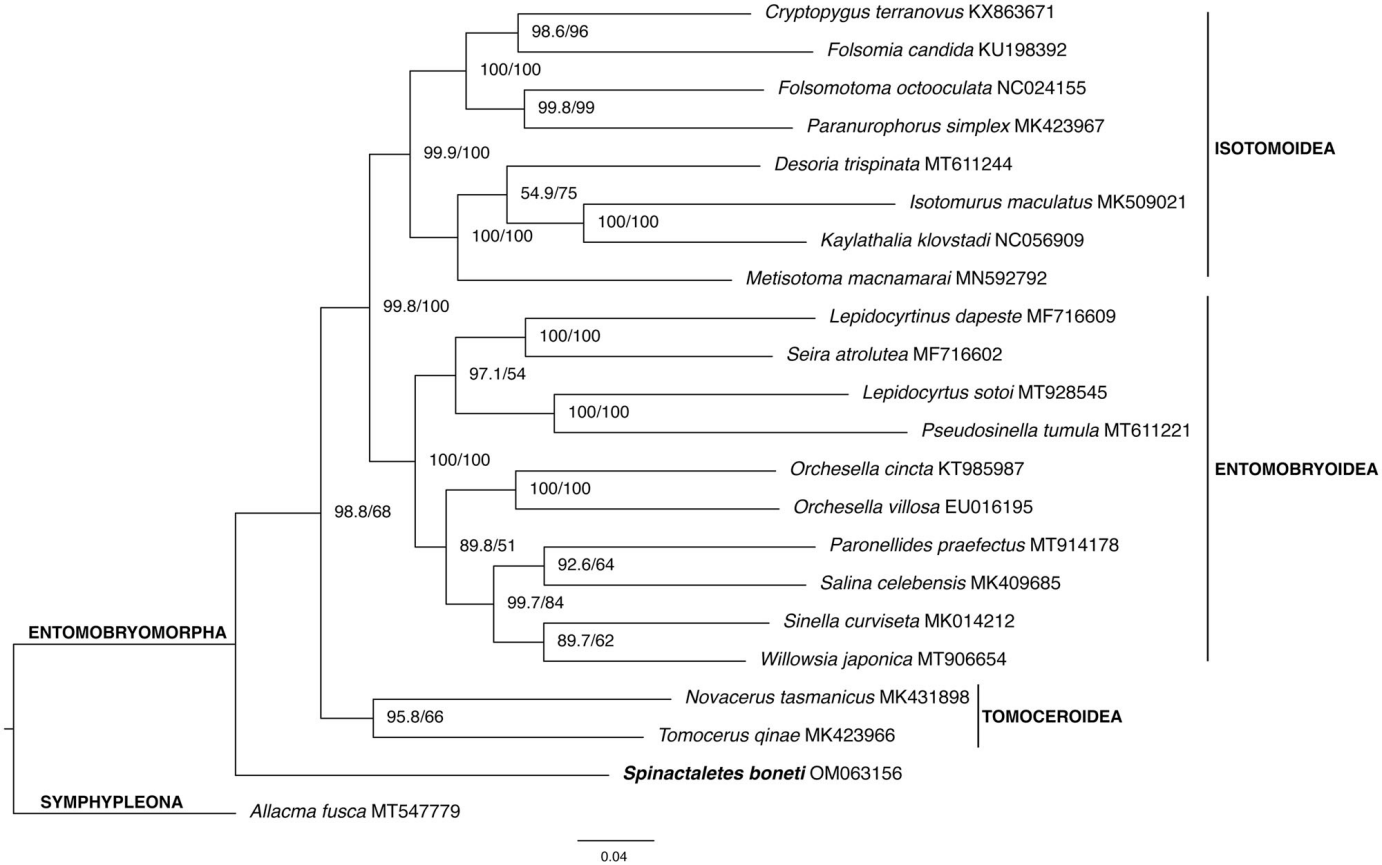
Phylogenetic placement of *Spinactaletes boneti* based on maximum likelihood inference. SH-aLRT (%) and ultrafast bootstrap (%) support values are indicated in each node and GenBank accession numbers are presented in the branches.

Previously to the phylogenetic analyses, mitogenomes sequences of 20 taxa of Entomobryomorpha and one of Symphypleona (outgroup) were downloaded from GenBank. All accession numbers are listed in Figure 1. The newly assembled mitogenome of *S. boneti* (14,873 bp) was included and the final dataset comprised 22 species. Nucleotide sequences of all 13 protein coding genes were aligned using MAGUS (Smirnov and Warnow 2021) and BMGE v1.12 (Criscuolo and Gribaldo 2010) was used to trim the alignments. FASconCAT-G v1.04 (Kück and Longo 2014) was used to concatenate the sequences and a phylogenetic matrix with a partition scheme was created. The final matrix comprised 8084 nucleotide sites. Bayesian phylogenetic inference was performed using PhyloBayes MPI Version 1.5a (Lartillot et al. 2013), with CAT-GTR model, two chains were run until the likelihood had satisfactorily converged (maxdiff< 0.1). Maximum Likelihood inference was performed using IQ-Tree v2.0.7 (Minh et al. 2020), ultrafast bootstrap 1000 replicates (Hoang et al. 2018), and SH-aLRT support. Model Finder (Subha Kalyaanamoorthy et al. 2017) selected the best partition scheme and GTR + F substitution model for the six partitions. The phylogenetic tree was visualized and edited in FigTree v1.4.2 (available on https://tree.bio.ed.ac.uk/software/figtree/). The resulting topology based on ML (Figure 1) suggested the position of *Spinataletes boneti* as a sister group of the remaining Entomobryomorpha species with moderate bootstrap support (68%). Bayesian analyses placed the new mitogenome in the same position but with a bit lower posterior probability support (0.63). Our result is in agreement with the study made by D’Haese (2003), based on 131 morphological characters from 67 Collembola taxa, which concluded that the springtails had a terrestrial edaphic origin with the semi-aquatic life representing a secondary specialization, not a primitive condition. Further analyses including more mitogenomes of Actaletidae and Coenaletidae taxa need to be carried out to verify our result, considering that the support value is not high enough to make a conclusion. Internal relationships of the superfamilies Entomobryoidea, Tomoceroidea, and Isotomoidea are not the focus of our study, so they are not discussed here.

## Data Availability

The mitogenome sequence data that support the findings of this study are openly available in GenBank of NCBI at (https://www.ncbi.nlm.nih.gov/) under the accession no. OM063156. The associated **BioProject**, **SRA**, and **Bio-Sample** numbers are PRJNA792924, SRR17381101, and SAMN24475446 respectively.
